# Difference in the relative biological effectiveness and DNA damage repair processes in response to proton beam therapy according to the positions of the spread out Bragg peak

**DOI:** 10.1186/s13014-017-0849-1

**Published:** 2017-07-03

**Authors:** Hidehiro Hojo, Takeshi Dohmae, Kenji Hotta, Ryosuke Kohno, Atsushi Motegi, Atsushi Yagishita, Hideki Makinoshima, Katsuya Tsuchihara, Tetsuo Akimoto

**Affiliations:** 1 Division of Radiation Oncology and Particle Therapy, National Cancer Center Hospital East, 6-5-1, Kashiwanoha, Kashiwa, Chiba, 277-8577 Japan; 2 High Energy Accelerator Research Organization, 1-1 Oho, Tsukuba, Ibaraki, 305-0801 Japan; 3 Department of Radiation Physics, The University of Texas M.D. Anderson Cancer Center, 1840 Old Spanish Trail, Houston, TX 77054 USA; 4 Division of Translational Research, EPOC, National Cancer Center, 6-5-1, Kashiwanoha, Kashiwa, Chiba, 277-8577 Japan

**Keywords:** Proton beam, Relative Biological Effectiveness, Linear Energy Transfer, Spread-Out Bragg Peak, DNA damage

## Abstract

**Background:**

Cellular responses to proton beam irradiation are not yet clearly understood, especially differences in the relative biological effectiveness (RBE) of high-energy proton beams depending on the position on the Spread-Out Bragg Peak (SOBP). Towards this end, we investigated the differences in the biological effect of a high-energy proton beam on the target cells placed at different positions on the SOBP, using two human esophageal cancer cell lines with differing radiosensitivities.

**Methods:**

Two human esophageal cancer cell lines (OE21, KYSE450) with different radiosensitivities were irradiated with a 235-MeV proton beam at 4 different positions on the SOBP (position #1: At entry; position #2: At the proximal end of the SOBP; position #3: Center of the SOBP; position #4: At the distal end of the SOBP), and the cell survivals were assessed by the clonogenic assay. The RBE_10_ for each position of the target cell lines on the SOBP was determined based on the results of the cell survival assay conducted after photon beam irradiation. In addition, the number of DNA double-strand breaks was estimated by quantitating the number of phospho-histone H2AX (γH2AX) foci formed in the nuclei by immunofluorescence analysis.

**Results:**

In regard to differences in the RBE of a proton beam according to the position on the SOBP, the RBE value tended to increase as the position on the SOBP moved distally. Comparison of the residual number of γH2AX foci at the end 24 h after the irradiation revealed, for both cell lines, a higher number of foci in the cells irradiated at the distal end of the SOPB than in those irradiated at the proximal end or center of the SOBP.

**Conclusions:**

The results of this study demonstrate that the RBE of a high-energy proton beam and the cellular responses, including the DNA damage repair processes, to high-energy proton beam irradiation, differ according to the position on the SOBP, irrespective of the radiosensitivity levels of the cell lines.

**Electronic supplementary material:**

The online version of this article (doi:10.1186/s13014-017-0849-1) contains supplementary material, which is available to authorized users.

## Background

Proton Beam Therapy (PBT) has been used clinically, with or without concurrent chemotherapy, for several cancers, including locally advanced non-small cell lung cancer or esophageal squamous cell carcinoma (ESCC) [1–5]. PBT is expected to be associated with reduced treatment-related toxicities, because of the unique physical characteristic of the proton beam, wherein the peak energy, represented by the so-called Bragg peak, is delivered just before the particles come to rest, with the energy declining rapidly thereafter [2, 6]. This indicates that in PBT, a higher dose can be delivered to the tumor, while keeping the dose to the surrounding normal tissues within an acceptable level. In the clinical setting, in order to obtain a homogeneous effect of proton beams against tumors, the position and width of the Bragg Peak are adjusted to the position and depth or width of the tumor, to create the so-called Spread-Out Bragg Peak (SOBP) and fit the peak to any type of planning target volume [6].

In the application of PBT to clinical cancer treatment, we routinely adopt a Relative Biological Effectiveness (RBE) of 1.1. The RBE is approximated based on reported results from in vivo and in vitro experiments using a hamster non-tumor cell line and rodent cancer cell lines [7–9]. Evaluation of the RBE using human malignant cell lines has been limited until now, and there are no reports yet of evaluation of the RBE using human cancer cell lines exhibiting different levels of radiosensitivity.

While the RBE has been regarded as being constant throughout the range of the SOBP, recent studies have suggested that the RBE of a 62-MeV proton beam increased as the position on the SOBP became more distal, along with increase of the linear energy transfer (LET) [10, 11]. However, clinically, the use of low-energy proton beams is usually limited to patients with superficially located tumors. In regard to differences in the RBE of a high-energy proton beam (156–230-MeV) according to the position on the SOBP, several studies have indicated that that the RBE is higher in the distal part of the SOBP; however, conflicting results have been reported, and no definite conclusions have been drawn yet [12–17]. Further investigations are therefore needed to clarify the exact differences between the observed and estimated values of the RBE of a high-energy proton beam, usually used for the treatment of deep-seated solid tumors such as ESCC, for various positions within the SOBP. In addition, the correlation between the RBE and LET of irradiation within the SOBP in human tumor cells is also not yet clearly understood [12].

The number of DNA double-strand breaks (DSBs), which are well-known to play a major role in cell killing, after proton beam irradiation, has been shown as a useful predictor of the tumor radiosensitivity [18, 19]. However, few studies have investigated the differences in the degree of DNA damage/ DNA repair processes according to the position on the SOBP. Furthermore, there are also no reports of the differences in the RBE or degree of DNA damage in response to high-energy proton beam irradiation in human cancer cell lines with differing radiosensitivity levels [15, 20].

Therefore, we investigated the differences in the RBE and the cellular responses, especially focusing on DNA-DSB repair processes, depending on the position of the target cells on the SOBP, in response to high-energy proton beam irradiation of the target cells using 2 ESCC cell lines with differing radiosensitivity levels.

## Methods

### Cell line and culture

The human esophageal cancer cell lines OE21 and KYSE450 were obtained from the cell banks of Public Health England (Salisbury, UK) and the National Institutes of Biomedical Innovation (Osaka, Japan), respectively, and used within 20 passages for the present experiments. Both the cell lines were authenticated for their identity by Short Tandem Repeat analysis. The cell lines were selected based on the results of preliminary experiments carried out using 6 cell lines that showed different sensitivities to a photon beam, i.e., the OE21 cells were moderately sensitive to a photon beam, while the KYSE450 cells showed a low sensitivity (Additional file 1: Figure S1). The cells were maintained in RPMI1640 medium (SIGMA-ALDRICH, Saint Louis, MO) containing filtered 10% fetal bovine serum (Biowest, Nuaillé, France). Both cell lines were incubated under 100% humidity in the presence of 5% CO_2_ at 37 °C.

### Photon and proton irradiations

The irradiation experiments were conducted at the National Cancer Center Hospital East (NCCNE). For the photon irradiation, both cell lines were irradiated with a 6-MV x-ray beam at the dose rate of 6 Gy/min, using Linac (Varian Medical Systems, Palo Alto, CA). For the proton irradiation, both cell lines were irradiated with a 235-MeV proton beam (Sumitomo Heavy Industry, Ltd., Tokyo, Japan). Proton beams dispersed by a double-scattering method were shaped down with a brass collimator to irradiate a field size of 20 cm × 20 cm. The depth of irradiation was precisely modulated by placing polyethylene plates of appropriate thickness, considering the water equivalent thickness of 3 mm calculated by the thickness of the flask or dish and medium on the incident side, at a position between the collimator and the samples. The field size was 15 cm × 20 cm and the flask surface dose homogeneity was ≥95%. To evaluate the difference in the cellular responses to the proton beam irradiation according to the position on the SOBP, the cells were irradiated at 4 different positions on the SOBP, which was 8 cm in width (position #1) At entry (49 mm); position # 2) Just proximal to the SOBP (107 mm); position #3) Center of the SOBP (135 mm); position #4) Distal end, at the fall-off, of the SOBP (163 mm), at the dose rate of 6 Gy/min. The dosimetry error in the absolute dose was estimated to be a maximum of 1% at the center of the SOBP. The dose distribution within the irradiation field was also examined and the error range was 1% to 5% at the center of the SOBP.

The cells were seeded onto 25-cm^2^ flasks (Corning, New York, NY) or 35-mm μ-dishes (Ibidi, Munich, Germany), and placed on polyethylene plates at the time of irradiation of the perpendicular proton or photon beam. All experiments were conducted in triplicate.

### Clonogenic assay

The OE21 and KYSE450 cells were seeded in triplicate onto 25-cm^2^ tissue culture flasks containing 5 ml of the culture medium at 400 to 1600 cells per flask, depending on the irradiation dose, and incubated for 24 h prior to the irradiation. The flasks were irradiated with the proton or photon beams and returned to the CO_2_ incubator at 37 °C. After 8 or 12 days, the colonies were fixed with 4% formalin solution and stained with 1% crystal violet. Colonies that contained more than 50 cells were counted, and the surviving fractions were calculated as the ratio of the plating efficiencies of the irradiated to unirradiated cells. Cell survival curves were fitted to a linear-quadratic model:

$$ \ln\ \left(\mathrm{S}\right)=-\upalpha \mathrm{D}-{\upbeta \mathrm{D}}^2, $$

where S represents the surviving fraction, and D represents the dose of radiation, α and βare adjustable parameters. Non-linear regression analysis was carried out using the ROOT software (https://root.cern.ch/). The RBE_10_ and RBE_37_ (doses required to obtain a reduction in the fraction of the surviving cells to 10% (D_10_) and 37% (D_37_), respectively), relative to that of a 6-MV photon beam, were calculated for each position on the SOBP [11].

The results derived from irradiation of the 6-MV photon beam were adopted as the reference RBE values, because the RBE has conventionally been calculated relative to the effect of a ^60^Co or 6-MV x-ray beam irradiation [7]. The LET profiles were calculated from analytical linear energy transfer calculations [21].

### Immunofluorescence analysis for γH2AX formation

Cells were seeded onto 35-mm μ-dishes at the density of 1.0 × 10^5^ to 4.0 × 10^5^ per dish for 24 h prior to the irradiation and cultured at 37 °C in the presence of 5% CO_2_; cells in the plateau phase of culture were used for all the experiments in this study. After the proton or photon beam irradiations, the cells were incubated for 0.5 h or 24 h, followed by washing thrice with phosphate-buffered saline (PBS) and fixing with 4% formaldehyde for 15 min at each time-point. They were then washed again with PBS three times for 5 min each and blocked with blocking buffer containing 1% filtered bovine serum albumin (BSA; Roche, Basel, Swiss) and 1% TritonX-100 (SIGMA-ALDRICH, Saint Louis, MO) in PBS, followed by incubation for 1 h at room temperature. Thereafter, the cells were incubated with anti-rabbit antibody directed against phospho-histone H2A.X Serine139 (Cell Signaling Technology, Danvers, MA) overnight at 4 °C. After incubation with the primary antibody, the cells were washed again three times with PBS, followed by the addition of blocking buffer containing goat anti-rabbit Alexa Fluor 488 IgG secondary antibody (Invitrogen, Waltham, MA) was added; the cell suspensions were then left to stand for 2 h at room temperature. Thereafter, the cells were washed again with PBS 3 times before the addition of mounting medium containing DAPI (4′6-diamidino-2-phenylindole VECTASHIELD, Vector Laboratories, Burlingame, CA). The experiments were performed in triplicate. Images were obtained under the Carl-Zeiss LSM710 confocal fluorescence microscope. We randomly examined six to nine microscopic fields for γH2AX (green) foci and nuclei stained with DAPI (blue).

The γH2AX foci in each cell were counted using the National Institutes of Health Image J software (https://imagej.nih.gov/ij/) and the mean number of foci per nucleus was calculated. More than 100–200 cells were evaluated after the irradiation.

### Statistical analysis

One-way analysis of variance with Tukey’s or Games-Howell’s test, depending on the assumption of equal variances, was used for evaluating the significance of differences if normal distribution of the data could be confirmed. Kruskal-Wallis’ test, followed by Dunn’s comparison was used if the data were non-normally distributed. *P* < 0.05 was set as representing significant difference in all the analyses.

## Results

### Depth dose, LET profile and proton dosimetry

**Fig. 1:**
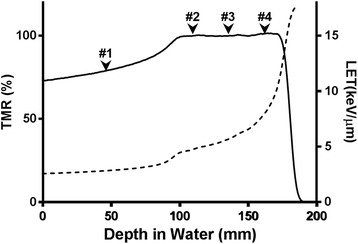
Depth dose and linear energy transfer (LET) profiles of the Spread-Out Bragg Peak (SOBP) of a 235-MeV proton beam. The solid line shows the tissue maximum ratio across the depth, and the dashed line indicates the LET

The depth in water and the LET profile of the 235-MeV proton beam at positions 1 to 4 of the SOBP are shown in Table 1 and Fig. 1. The LET data of the 6-MV x-ray beam were referenced from published data [22]. The LET increased steeply with increasing depth on the SOBP and became maximal at the distal end of the SOBP, while remaining stable at beam entry. The LET was determined to be 5.64 keV/μm at the center of the SOBP (position #3) and 8.14 keV/μm at the distal end of the SOBP (position #4) (Table 1). The α value of the proton beam irradiation in the OE21 cells was higher than that of the 6MV photon beam, while the β values were not significantly different. For the case of the KYSE450 cells, the α value of proton beam irradiation within the SOBP tended to be higher than that of the 6MV photon beam irradiation.

**Table 1:** LET and survival parameters for OE21 and KYSE450

OE21	LET (keV / μm)	α (Gy^−1^)	β (Gy^−2^)	α/β (Gy)	D_10_ (Gy)	D_37_ (Gy)	RBE_10_	RBE_37_
6MV X-ray	2.36	0.06 ± 0.03	0.06 ± 0.01	0.88	5.63 ± 0.14	3.55 ± 0.13		
#1	2.85	0.17 ± 0.03	0.05 ± 0.01	3.23	5.36 ± 0.29	3.07 ± 0.05	1.06 ± 0.04	1.16 ± 0.03
#2	4.65	0.23 ± 0.03	0.05 ± 0.01	4.37	4.80 ± 0.10	2.68 ± 0.08	1.17 ± 0.02	1.33 ± 0.04
#3	5.64	0.16 ± 0.07	0.07 ± 0.02	2.16	4.63 ± 0.15	2.71 ± 0.06	1.22 ± 0.03	1.31 ± 0.03
#4	8.14	0.24 ± 0.05	0.06 ± 0.01	4.05	4.56 ± 0.11	2.57 ± 0.16	1.24 ± 0.03	1.40 ± 0.06
KYSE450	LET (keV / μm)	α	β	α/β	D_10_	D_37_	RBE_10_	RBE_37_
6MV X-ray	2.36	0.06 ± 0.02	0.02 ± 0.00	3.40	9.55 ± 0.30	5.81 ± 0.30		
#1	2.85	0.05 ± 0.03	0.02 ± 0.00	2.38	9.25 ± 0.13	5.70 ± 0.16	1.03 ± 0.02	1.02 ± 0.04
#2	4.65	0.08 ± 0.04	0.02 ± 0.01	3.81	9.06 ± 0.20	5.35 ± 0.22	1.06 ± 0.02	1.09 ± 0.04
#3	5.64	0.07 ± 0.03	0.03 ± 0.01	2.64	7.98 ± 0.18	4.82 ± 0.07	1.20 ± 0.03	1.21 ± 0.04
#4	8.14	0.09 ± 0.03	0.03 ± 0.00	3.11	7.68 ± 0.03	4.61 ± 0.15	1.24 ± 0.02	1.27 ± 0.05

*LET* linear energy transfer, *D*_*10*_ dose required to reduce the fraction of surviving cells to 10%, *D*_*37*_ dose required to reduce the fraction of surviving cells to 37%, *RBE*_*10*_ relative biological effectiveness at the survival dose of 10%, *RBE*_*37*_ relative biological effectiveness at the survival dose of 37%

### Cell survival response curve in the OE21 and KYSE450 cells

**Fig. 2:**
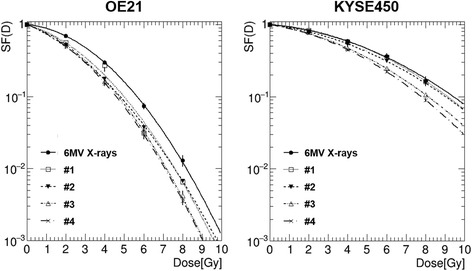
Survival curves of the OE21 and KYSE450 cells following photon- beam and proton-beam irradiation at 4 different positions on the SOBP. Error bars indicate the standard error of the mean

The survival curve after photon irradiation showed that the shoulder of the survival curves was larger for the OE21 cells (α = 0.06 ± 0.03, β = 0.06 ± 0.01) than for the KYSE450 cells (α = 0.06 ± 0.02, β = 0.02 ± 0.00) (Fig. 2). The survival curves for the OE21 and KYSE450 cells irradiated with the proton beam at the 4 specified positions on the SOBP are also shown in Fig. 2. The surviving fraction of the OE21 cells following irradiation at the dose of 8 Gy was significantly different between position #1 and position #4 (*p* = 0.017), and also between position #2 and position #4 (*p* = 0.012). The surviving fraction of the KYSE450 cells following irradiation at the dose of 6 Gy was significantly between position #1 and position #3 or position #4 (*p* = 0.002), and between position #2 and position #3 or position #4 (*p* = 0.031); furthermore, the surviving fraction of the cells following irradiation at 8 Gy was also significantly different between position #1 and position #3 or position #4 (*p* = 0.003), and between position #2 and position #3 or position #4 (*p* = 0.007) (Additional file 2: Figure S2). These results and the radiosensitivity parameters, including the RBE, are summarized in Table 1. Although there were no significant differences in the RBE_10_ among position #2, position #3 and position #4 on the SOBP in the OE21 cells, there were significant differences in the RBE_10_ between position #1 and the other specified positions on the SOBP (#2, *p* = 0.04; #3, *p* = 0.004; #4, *p* = 0.001), and a trend towards a higher RBE than the routinely used fixed RBE value of 1.1 at the distal end of the SOBP. The differences in the RBE_10_ and RBE_37_ in the OE21 cells between position #2 and position #4 were 6.0% and 5.4%, respectively. The differences in the RBE_10_ among the positions on the SOBP were also significant (position #2 vs. position #3, *p* = 0.04; position #2 vs. position #4, *p* = 0.04) in the KYSE450 cells. Furthermore, the differences in the RBE_10_ and RBE_37_ in the KYSE450 cells between position #2 and position #4 were 24% and 17%, respectively. Consistent with these findings, the RBE_10_ and RBE_37_ of both cell lines were significantly different between beam entry and positions on the SOBP, and the differences among positions within the SOBP were also over 5%.

### Correlation between the LET and RBE

**Fig. 3:**
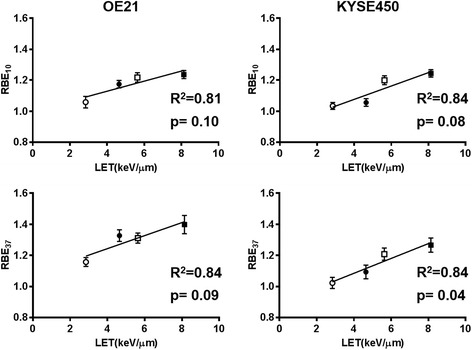
Correlation between the RBE and the LET. The clear and filled circles show the results for positions #1 and #2 on the SOBP, respectively. The clear and filled squares show the results for positions #3 and #4 on the SOBP, respectively. Error bars indicate the standard error of the mean. A strong positive correlation between the RBE and LET was observed in both the OE21 and KYSE450 cells

Figure 3 shows the correlation between the LET and RBE for both cell lines. There was a trend towards a positive correlation between the LET and RBE_10_/RBE_37_ for both cell lines (OE21: R^2^ = 0.81/R^2^ = 0.91; KYSE450: R^2^ = 0.84/R^2^ = 0.92), while the correlation between the RBE_37_ and LET in the KYSE450 cells (*p* = 0.04) was statistically significant.

### Differences in the DNA-DSB and repair kinetics for positions along the SOBP

Additional file 3: Figure S3 shows the results of the immunofluorescence analysis for γH2AX foci. Both cell lines were irradiated with the proton beam at each of the four positions on the SOPB, at the dose of 8 Gy. In the preliminary study, we confirmed the dose-response between the number of gamma-H2AX foci and doses up to 8 Gy. Although there was an increase in the number of microscopically visible γH2AX foci at 0.5 h after the irradiation in both cell lines for all positions, the number of residual foci at 24 h after the irradiation differed in both the cell lines according to the position on the SOBP.

**Fig. 4:**
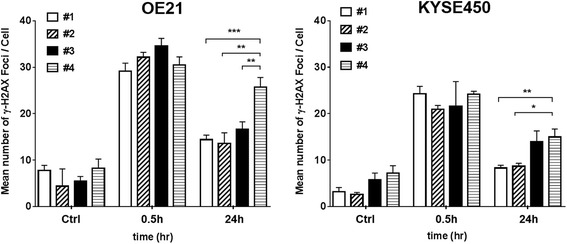
γH2AX analysis after 8 Gy proton beam irradiation. The numbers of foci formed per cell were counted at 0.5 and 24 h after the irradiation in the OE21 and KYSE450 cells. Error bars represent the standard error of the mean from 3 to 4 experiments. (*p < 0.05, **p < 0.01, ***p < 0.001)

Figure 4 shows the number of γH2AX foci in individual cells that were not irradiated and in those that were exposed to 8 Gy radiation at position #1, position #2, position #3 and position #4. At 0.5 h, no significant differences in the number of foci were observed among the positions. However, at 24 h after the irradiation, the number of residual foci in the OE21 cells was significantly higher for position #4 than for position #1, position #2 or position #3 (*p* = 0.000, *p* = 0.002, and *p* = 0.007, respectively). A similar trend was noted in the KYSE450 cells. There were significant differences in the number of γH2AX foci in the cells at 24 h after irradiation between position #1 and position #4 and between position #2 and position #4 (*p* = 0.003, *p* = 0.019).

**Fig. 5:**
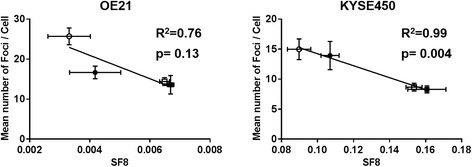
Correlation between the mean number of foci per cell at 24 h after the irradiation and the survival fraction after 8 Gy proton beam irradiation (SF8). The clear and filled circles show the irradiated #1 and #2, respectively. The clear and filled squares show the results for position #3 and position #4, respectively. Error bars indicate the standard error of the mean. A strong negative correlation between the mean number of foci formed per cell and the SF8 was observed in the OE21 cells, and a significant correlation was also found in the KYSE450 cells (*p* = 0.004)

The correlation between the surviving fraction after irradiation at 8 Gy (SF8) and the residual number of γH2AX foci/cell at 24 h after irradiation is shown in Fig. 5. A significant correlation between the SF8 and residual number of foci/cell at 24 h after the irradiation was observed in both OE21 and KYSE450 cells (R^2^ = 0.76 and R^2^ = 0.99, respectively). Additional file 4: Figure S4 shows the correlation between the LET and the residual number of γH2AX foci/cell at 24 h: a good correlation between the two parameters was observed in both cell lines.

## Discussion

The RBE tended to be higher at the distal end of the SOBP than that at the proximal portion of the SOBP, irrespective of the radiosensitivity of the cell lines used. In addition, the amount of unrepaired double-stranded DNA breaks, as assessed by the number of γH2AX foci formed in the cells, at 24 h after irradiation was higher for irradiation at the distal end of the SOBP. Our findings indicate that the RBE as well as the cellular responses, including DNA damage repair processes, in response to proton beam irradiation differed according to the position on the SOBP.

The RBE of proton beam irradiation against two ESCC cell lines of differing radiosensitivities in this study differed according to the position on the SOBP, and varied in the range of 1.03 to 1.40. Previous reports have shown differences in the RBE, ranging from 1.3 to 2.8, according to the depth, namely, position on the SOBP, for lower-energy proton beams (62–87-MeV) [10, 12, 13]. However, inconsistent results have been reported from previous studies for the case of high-energy proton beams (200–230-MeV), which are widely used for the clinical treatment of solid tumors. Britten et al., reported for the case of a 200-MeV proton beam, that the range of the RBE at the distal 3 points on the SOBP was small (LET: 7.8–13.6 keV/μm) [12]. Calugaru et al. showed, using a 201-MeV proton beam, that the RBE was almost similar throughout the course of the SOBP [13]. A recent study, in which 160 (LET: proximal of SOBP; 2.05 keV/μm, distal of SOBP; 3.2 keV/μm) and 230-MeV (LET: proximal of SOBP; 1.95 keV/μm, distal of SOBP; 2.95 keV/μm) proton beams were used, showed that the RBE at the distal end of the SOBP was about 6% higher as compared to that at the proximal portion of the SOBP [17]. Actually, the results of this study conducted using a 235-MeV proton beam demonstrated that the RBE against cells at a depth corresponding to the distal end of the SOBP was significantly higher than that against cells at a depth corresponding to the proximal portion of the SOBP (range 5.4–24%) (LET: at the proximal portion of the SOBP, 4.65 keV/μm; at the distal portion of the SOBP, 8.14 keV/μm), irrespective of the radiosensitivity levels of the ESCC cell lines used.

Evaluation of the differences in the RBE depending on the position along the SOBP in previously reported studies has mainly been conducted using rodent cells and human malignant tumor cells (human salivary gland tumor (HSG) cells, human cervical carcinoma (HeLa) cells, head and neck squamous carcinoma (SQ20B) cells, and human melanoma (HTB140) cells [10, 12–14, 16, 23]. However, there have been no studies of the influence of the radiosensitivity levels of the cells on the differences in the RBE of a proton beam according to the position on the SOBP. Therefore, we consider that the results of this study provide valuable and useful information regarding variations of the RBE depending on the position of the irradiation target on the SOBP in clinical settings, especially for deep-seated cancers, such as esophageal cancer.

Differences in the RBE for irradiation targets in different positions along the SOBP would also be expected to influence the cellular responses to the proton beam. In explaining the mechanism underlying the correlation between the RBE and the cellular responses, LET has an important role. LET has been demonstrated to increase steeply at the distal end of the SOBP [11, 20, 24]. Consistent with the reports of several previous investigators in regard to the existence of a significant relationship between the RBE and LET [11, 12, 25], the current study also showed a trend towards a positive correlation between the RBE and LET for both cell lines, despite their different sensitivities to the proton beam.

When DSBs occur in cells exposed to photon- or proton-beam irradiation, formation of γH2AX foci represents one of the earliest events of the DNA damage repair process; these foci appear within minutes and reach their peak number at 0.5 h [26]. In the current study, the position of the irradiation target on the SOBP had no influence on the number of γH2AX foci counted at 0.5 h after the irradiation, suggesting that the number of DNA-DSBs does not differ according to the position on the SOBP. DNA-DSBs have considered to be induced linearly, with a yield of approximately 20–40 per cell nucleus per Gy. Under this presumption, the number of foci formed after irradiation of 8 Gy was relatively low. However, there are discrepancies among published data on γH2AX foci formation after irradiation, as several factors, such as differences in the cell types, study protocols and reagents, can influence the early kinetics of foci formation and loss [27]. The mean number of γH2AX foci per cell observed at 25 min after proton or photon irradiation at 1 Gy in A549 cells is reported to be 5–10 [28]. In addition, at higher irradiation doses, the foci tend to become large, probably due to the overlapping and merging of different foci into larger areas, as shown in Additional file 3: Figure S3. Therefore, we consider that our results on the number of foci formation at 0.5 h after irradiation of 8 Gy exactly consistent with the trends of changes in the number of γH2AX foci formed after irradiation.

In contrast, the residual number of γH2AX foci at 24 h after the irradiation was significantly higher for position #4 (distal end) of the SOBP in both cell lines.

The residual number of γH2AX foci has been interpreted as being reflective of persistent unrepaired DNA damage and as a predictor of the tumor radiosensitivity [18, 19, 29]. In a recent investigation of human lung carcinoma cell lines (A549) and V79 cells, the numbers of γH2AX and phosphorylated p53 binding protein 1 (53BP1) foci at 1 h and 12 h after exposure to 156.7-MeV to 182.8-MeV proton beam irradiation was lower for the irradiation target at the center of the SOBP than that for that at the distal end of the SOBP [15]. Chaudhary et al. demonstrated that the number of 53BP1 foci in AG015822 cells (normal human skin fibroblasts) following irradiation with a 60-MeV proton beam at the distal end of the SOBP was significantly increased at 24 h after the irradiation [20]. The degree of DNA damage and the repair processes in the two ESCC cell lines with differing radiosensitivities following irradiation with a 235-MeV proton beam were almost similar to those reported for other cell lines irradiated with 60–182.8-MeV proton beams [15, 20]. In addition, our study also demonstrated that the number of γH2AX foci at 24 h after proton beam irradiation might serve as a predictor of the radiosensitivity of the ESCC cell lines, because a significant correlation was observed between the SF8 and residual number of γH2AX foci at 24 h after the proton beam irradiation.

The residual number of γH2AX foci is usually increased by high-LET irradiation. Bracalente et al. showed that the residual number of γH2AX foci at 6 h after high-LET irradiation was higher than that after γ-ray irradiation in a Chinese hamster ovary cell line [30]. Antonelli et al. also revealed that the higher the LET, the longer the γH2AX foci persist, as compared to that following irradiation with γ-rays [31]. In this study, we found a positive correlation between the LET and the residual number of γH2AX foci at 24 h after irradiation, irrespective of the radiosensitivity level of the ESCC cell lines used in the study.

In the clinical setting, we often select the physical proton dose based on the RBE of 1.1, assuming homogeneity along all positions of the SOBP. However, our current study demonstrated that the RBE differed according to the position on the SOBP. Moreover, the differences in the RBE among the positions on the SOBP in both cell lines were 5% or more (differences in the RBE exceeding 5% would affect the clinical outcomes, including the efficacy of local control or toxicities). Thus, it may be necessary to take into consideration an inhomogeneous distribution of the RBE in the range of the SOBP for treatment planning in patients scheduled to undergo PBT. Further clinical studies are needed to clarify how the inhomogeneity of the RBE within the range of the SOBP would affect the clinical outcomes.

Our study had the following limitations. First, we evaluated only 2 cancer cell lines. The RBE and cellular responses to proton beam irradiation could differ depending on the cell line used. Therefore, caution should be exercised in extrapolating the results of this study to other type of tumors or normal cells. Second, it seemed that the number of γH2AX foci at 0.5 h after irradiation of 8Gy using the National Institutes of Health Image J software was low. Thus, further analysis using another technique, such as flow cytometry, would be needed. Third, the current study was performed in vitro. In order to apply the results to clinical treatment, investigations in vivo would be needed, because the microenvironments of tumors and/or normal tissues could be expected to affect the RBE and cellular responses to proton beam irradiation. Thus, further investigations are warranted to confirm the validity of the results of this study. In addition, while PBT has been used with concurrent chemotherapy for locally advanced cancers, such as locally advanced non-small cell lung cancer, in clinical practice, there are few radiobiological reports regarding the effect of PBT used in combination with chemotherapy. Therefore, we should clarify the influence of differences in the radiosensitizing effects of the chemotherapeutic agents used on the differences in the effects of proton beam irradiation depending on the position on the SOBP.

## Conclusions

In this study, we demonstrated that the RBE of a high-energy proton beam and the cellular responses, including the DNA damage repair processes, to irradiation with a high-energy proton beam, differed according to the position on the SOBP, regardless of the radiosensitivity levels of the cells, in two human cancer cell lines.

## Additional files

Additional file 1: Figure S1. Survival curves of 6 different esophageal squamous cell carcinoma cell lines irradiated with a 150-kV photon beam in preliminary experiments. Each experiment was conducted in triplicate. Error bars indicate the standard error of the mean. (TIFF 343 kb)

Additional file 2: Figure S2. Surviving fraction following irradiation at 6 Gy and 8 Gy in the OE21 and KYSE450 cells. Error bars indicate the standard error of the mean. (*p < 0.05, **p < 0.01, ***p < 0.001) (TIFF 337 kb)

Additional file 3: Figure S3. Confocal images of OE21 and KYSE450 cells obtained after 0.5 h and 24 h following irradiation with a proton beam at each position of the SOBP. Cells were stained with anti-γH2AX (green fluorescence dye) antibody and DAPI (blue). (TIFF 2421 kb)

Additional file 4: Figure S4. Correlation with the mean number of foci per cell at 24 h after the irradiation and the linear energy transfer (LET). The clear and filled circles show the results for position #1 and position #2, respectively. The clear and filled squares show the results for position #3 and position #4, respectively. Error bars indicate the standard error of the mean. A strong positive correlation between the mean number of foci formed per cell and the LET was observed in both the OE21 and KYSE450 ells (R^2^ = 0.81 and R^2^ = 0.79, respectively). (TIFF 225 kb)

## Abbreviations

53BP1: Phosphorylated p53 binding protein 1
DNA: Deoxyribonucleic acid
DSBs: Double-strand breaks
ESCC: Esophageal squamous cell carcinoma
LET: Linear energy transfer
NCCHE: National Cancer Center Hospital East
PBT: Proton beam therapy
RBE: Relative biological effectiveness
SF8: Surviving fraction after irradiation at 8 Gy
SOBP: Spread-Out Bragg Peak
γH2AX: phospho-histone H2AX

## References

[1] Chang JY, Komaki R, Lu C, Wen HY, Allen PK, Tsao A, Gillin M, Mohan R, Cox JD (2011). Phase 2 study of high-dose proton therapy with concurrent chemotherapy for unresectable stage III nonsmall cell lung cancer. Cancer.

[2] Lin SH, Komaki R, Liao Z, Wei C, Myles B, Guo X, Palmer M, Mohan R, Swisher SG, Hofstetter WL (2012). Proton beam therapy and concurrent chemotherapy for esophageal cancer. Int J Radiat Oncol Biol Phys.

[3] Oshiro Y, Mizumoto M, Okumura T, Hashimoto T, Fukumitsu N, Ohkawa A, Kanemoto A, Hashii H, Ohno T, Sakae T (2012). Results of proton beam therapy without concurrent chemotherapy for patients with unresectable stage III non-small cell lung cancer. J Thorac Oncol.

[4] Oshiro Y, Okumura T, Kurishima K, Homma S, Mizumoto M, Ishikawa H, Onizuka M, Sakai M, Goto Y, Hizawa N (2014). High dose concurrent chemo-proton therapy for Stage III NSCLC: preliminary results of a Phase II study. J Radiat Res.

[5] Takada A, Nakamura T, Takayama K, Makita C, Suzuki M, Azami Y, Kato T, Tsukiyama I, Hareyama M, Kikuchi Y (2016). Preliminary treatment results of proton beam therapy with chemoradiotherapy for stage I-III esophageal cancer. Cancer Med.

[6] Romesser PB, Cahlon O, Scher E, Zhou Y, Berry SL, Rybkin A, Sine KM, Tang S, Sherman EJ, Wong R, Lee NY (2016). Proton beam radiation therapy results in significantly reduced toxicity compared with intensity-modulated radiation therapy for head and neck tumors that require ipsilateral radiation. Radiother Oncol.

[7] Paganetti H (2014). Relative biological effectiveness (RBE) values for proton beam therapy. Variations as a function of biological endpoint, dose, and linear energy transfer. Phys Med Biol.

[8] Paganetti H, Niemierko A, Ancukiewicz M, Gerweck LE, Goitein M, Loeffler JS, Suit HD (2002). Relative biological effectiveness (RBE) values for proton beam therapy. Int J Radiat Oncol Biol Phys.

[9] Tommasino F, Durante M (2015). Proton radiobiology. Cancers (Basel).

[10] Petrovic I, Ristic-Fira A, Todorovic D, Koricanac L, Valastro L, Cirrone P, Cuttone G (2010). Response of a radioresistant human melanoma cell line along the proton spread-out Bragg peak. Int J Radiat Biol.

[11] Chaudhary P, Marshall TI, Perozziello FM, Manti L, Currell FJ, Hanton F, McMahon SJ, Kavanagh JN, Cirrone GA, Romano F (2014). Relative biological effectiveness variation along monoenergetic and modulated Bragg peaks of a 62-MeV therapeutic proton beam: a preclinical assessment. Int J Radiat Oncol Biol Phys.

[12] Britten RA, Nazaryan V, Davis LK, Klein SB, Nichiporov D, Mendonca MS, Wolanski M, Nie X, George J, Keppel C (2013). Variations in the RBE for cell killing along the depth-dose profile of a modulated proton therapy beam. Radiat Res.

[13] Calugaru V, Nauraye C, Noel G, Giocanti N, Favaudon V, Megnin-Chanet F (2011). Radiobiological characterization of two therapeutic proton beams with different initial energy spectra used at the Institut Curie Proton Therapy Center in Orsay. Int J Radiat Oncol Biol Phys.

[14] Iwata H, Ogino H, Hashimoto S, Yamada M, Shibata H, Yasui K, Toshito T, Omachi C, Tatekawa K, Manabe Y (2016). Spot scanning and passive scattering proton therapy: relative biological effectiveness and oxygen enhancement ratio in cultured cells. Int J Radiat Oncol Biol Phys.

[15] Maeda K, Yasui H, Yamamori T, Matsuura T, Takao S, Suzuki M, Matsuda A, Inanami O, Shirato H (2016). A nucleoside anticancer drug, 1-(3-C-Ethynyl-beta-D-Ribo-Pentofuranosyl)cytosine, induces depth-dependent enhancement of tumor cell death in Spread-Out Bragg Peak (SOBP) of proton beam. PLoS One.

[16] Matsumoto Y, Matsuura T, Wada M, Egashira Y, Nishio T, Furusawa Y (2014). Enhanced radiobiological effects at the distal end of a clinical proton beam: in vitro study. J Radiat Res.

[17] Wouters BG, Skarsgard LD, Gerweck LE, Carabe-Fernandez A, Wong M, Durand RE, Nielson D, Bussiere MR, Wagner M, Biggs P (2015). Radiobiological intercomparison of the 160 MeV and 230 MeV proton therapy beams at the Harvard Cyclotron Laboratory and at Massachusetts General Hospital. Radiat Res.

[18] Olive PL (2011). Retention of gammaH2AX foci as an indication of lethal DNA damage. Radiother Oncol.

[19] Taneja N, Davis M, Choy JS, Beckett MA, Singh R, Kron SJ, Weichselbaum RR (2004). Histone H2AX phosphorylation as a predictor of radiosensitivity and target for radiotherapy. J Biol Chem.

[20] Chaudhary P, Marshall TI, Currell FJ, Kacperek A, Schettino G, Prise KM (2016). Variations in the processing of DNA double-strand breaks along 60-MeV therapeutic proton beams. Int J Radiat Oncol Biol Phys.

[21] Wilkens JJ, Oelfke U (2003). Analytical linear energy transfer calculations for proton therapy. Med Phys.

[22] Okamoto H, Kanai T, Kase Y, Matsumoto Y, Furusawa Y, Fujita Y, Saitoh H, Itami J, Kohno T (2011). Relation between lineal energy distribution and relative biological effectiveness for photon beams according to the microdosimetric kinetic model. J Radiat Res.

[23] Kanemoto A, Hirayama R, Moritake T, Furusawa Y, Sun L, Sakae T, Kuno A, Terunuma T, Yasuoka K, Mori Y (2014). RBE and OER within the spread-out Bragg peak for proton beam therapy: in vitro study at the Proton Medical Research Center at the University of Tsukuba. J Radiat Res.

[24] Calugaru V, Nauraye C, Cordelieres FP, Biard D, De Marzi L, Hall J, Favaudon V, Megnin-Chanet F (2014). Involvement of the Artemis protein in the relative biological efficiency observed with the 76-MeV proton beam used at the Institut Curie Proton Therapy Center in Orsay. Int J Radiat Oncol Biol Phys.

[25] Paganetti H, Olko P, Kobus H, Becker R, Schmitz T, Waligorski MP, Filges D, Muller-Gartner HW (1997). Calculation of relative biological effectiveness for proton beams using biological weighting functions. Int J Radiat Oncol Biol Phys.

[26] Ivashkevich A, Redon CE, Nakamura AJ, Martin RF, Martin OA (2012). Use of the gamma-H2AX assay to monitor DNA damage and repair in translational cancer research. Cancer Lett.

[27] Rothkamm K, Horn S (2009). gamma-H2AX as protein biomarker for radiation exposure. Ann Ist Super Sanita.

[28] Fontana AO, Augsburger MA, Grosse N, Guckenberger M, Lomax AJ, Sartori AA, Pruschy MN (2015). Differential DNA repair pathway choice in cancer cells after proton- and photon-irradiation. Radiother Oncol.

[29] Lobrich M, Shibata A, Beucher A, Fisher A, Ensminger M, Goodarzi AA, Barton O, Jeggo PA (2010). gammaH2AX foci analysis for monitoring DNA double-strand break repair: strengths, limitations and optimization. Cell Cycle.

[30] Bracalente C, Ibanez IL, Molinari B, Palmieri M, Kreiner A, Valda A, Davidson J, Duran H (2013). Induction and persistence of large gammaH2AX foci by high linear energy transfer radiation in DNA-dependent protein kinase-deficient cells. Int J Radiat Oncol Biol Phys.

[31] Antonelli F, Campa A, Esposito G, Giardullo P, Belli M, Dini V, Meschini S, Simone G, Sorrentino E, Gerardi S (2015). Induction and repair of DNA DSB as revealed by H2AX phosphorylation foci in human fibroblasts exposed to low- and high-LET radiation: relationship with early and delayed reproductive cell death. Radiat Res.

