# Supplementary figures and images for: Difference in the relative biological effectiveness and DNA damage repair processes in response to proton beam therapy according to the positions of the spread out Bragg peak
Source: Radiat Oncol. 2017 Jul 3;12:111. doi: 10.1186/s13014-017-0849-1 (PMC5494883; doi:10.1186/s13014-017-0849-1)

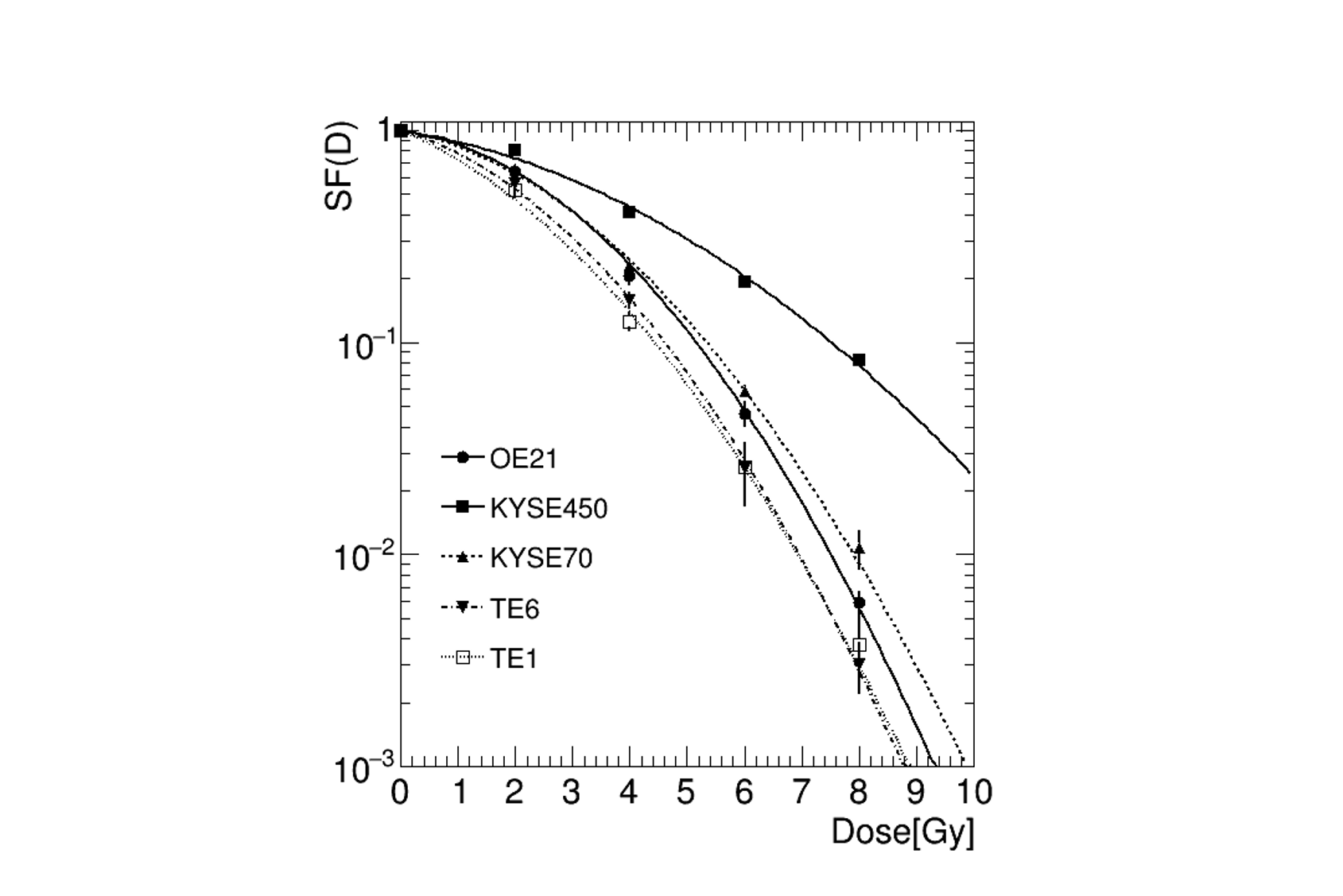

Supplement: Supplementary file 1 — Survival curves of 6 different esophageal squamous cell carcinoma cell lines irradiated with a 150-kV photon beam in preliminary experiments. Each experiment was conducted in triplicate. Error bars indicate the standard error of the mean. (TIFF 343 kb) [file 13014_2017_849_MOESM1_ESM.tif]

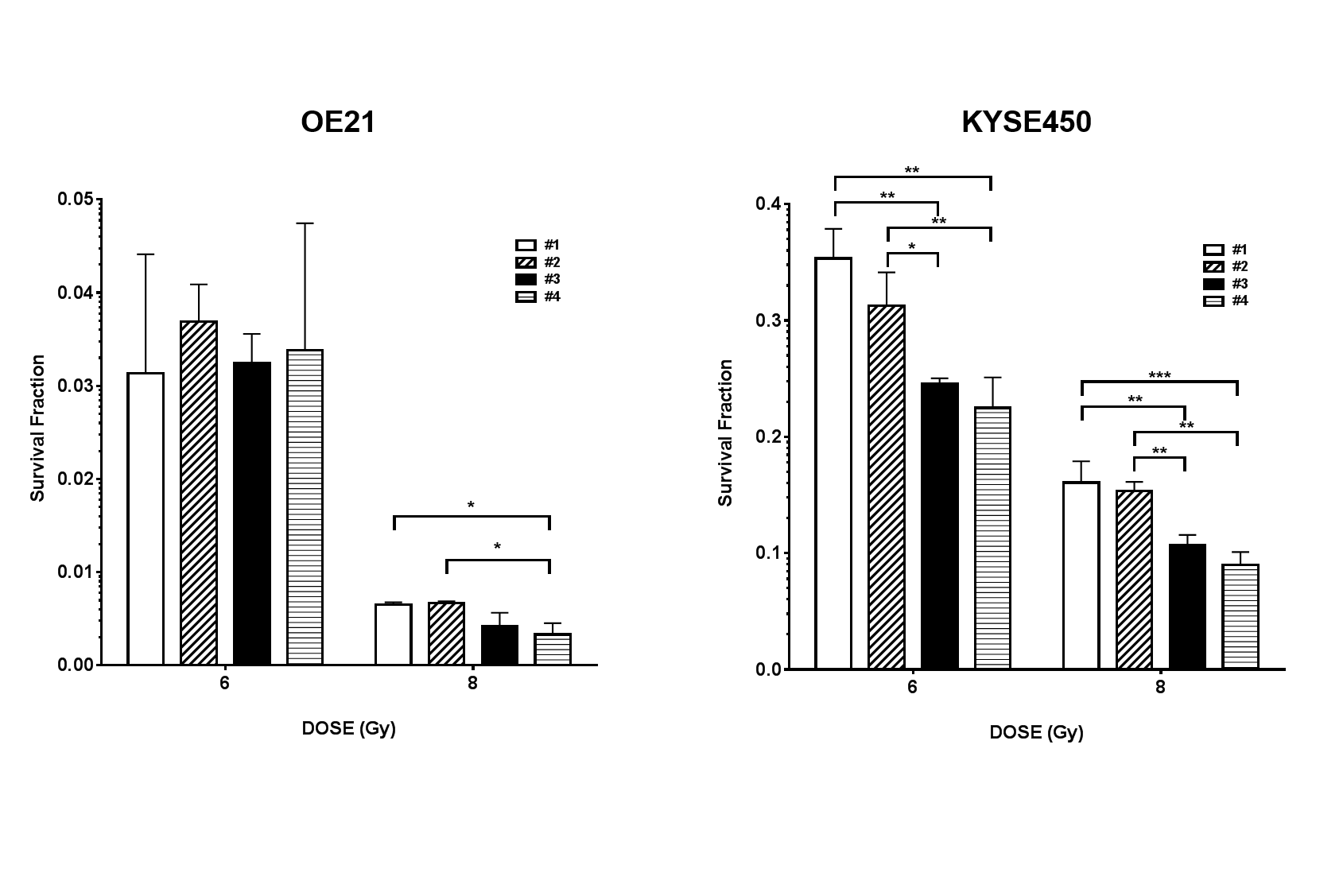

Supplement: Supplementary file 2 — Surviving fraction following irradiation at 6 Gy and 8 Gy in the OE21 and KYSE450 cells. Error bars indicate the standard error of the mean. (*p < 0.05, **p < 0.01, ***p < 0.001) (TIFF 337 kb) [file 13014_2017_849_MOESM2_ESM.tif]

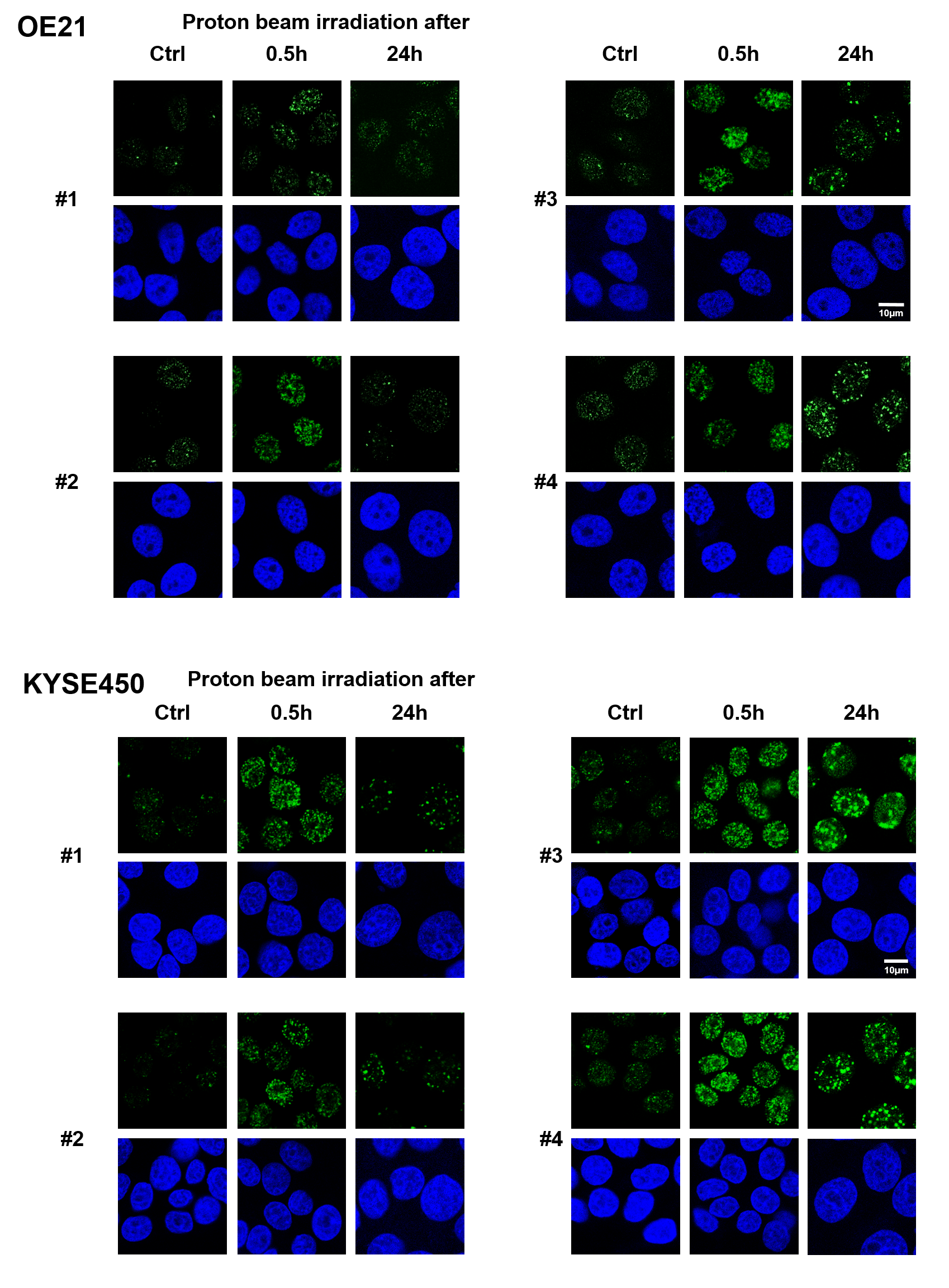

Supplement: Supplementary file 3 — Confocal images of OE21 and KYSE450 cells obtained after 0.5 h and 24 h following irradiation with a proton beam at each position of the SOBP. Cells were stained with anti-γH2AX (green fluorescence dye) antibody and DAPI (blue). (TIFF 2421 kb) [file 13014_2017_849_MOESM3_ESM.tif]

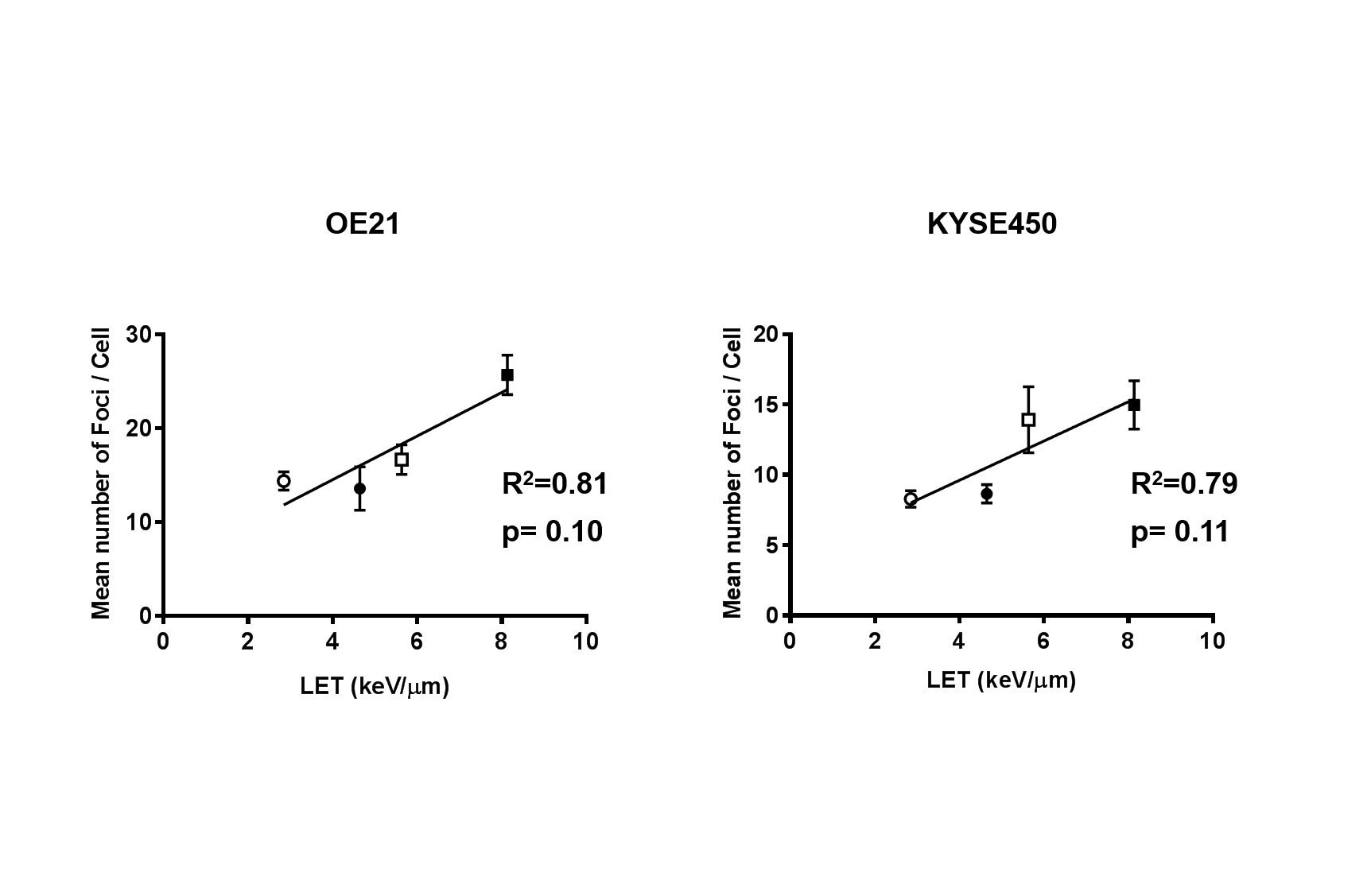

Supplement: Supplementary file 4 — Correlation with the mean number of foci per cell at 24 h after the irradiation and the linear energy transfer (LET). The clear and filled circles show the results for position #1 and position #2, respectively. The clear and filled squares show the results for position #3 and position #4, respectively. Error bars indicate the standard error of the mean. A strong positive correlation between the mean number of foci formed per cell and the LET was observed in both the OE21 and KYSE450 ells (R2 = 0.81 and R2 = 0.79, respectively). (TIFF 225 kb) [file 13014_2017_849_MOESM4_ESM.tif]
